# The Usefulness of 2-[18F]FDG PET or PET/CT in Extranodal Natural Killer/T-Cell Lymphoma: A Systematic Review and Meta-Analysis

**DOI:** 10.3390/jcm14134582

**Published:** 2025-06-27

**Authors:** Domenico Albano, Carlo Rodella, Alessandra Tucci, Giorgio Treglia, Francesco Bertagna, Arturo Chiti, Federico Fallanca

**Affiliations:** 1 Nuclear Medicine, ASST Spedali Civili Brescia, 25123 Brescia, Italy; francesco.bertagna@unbs.it; 2 Nuclear Medicine, Università degli Studi di Brescia, 25125 Brescia, Italy; 3 Health Physics Department, ASST Spedali Civili di Brescia, 25123 Brescia, Italy; carlo.rodella@asst-spedalicivili.it; 4 Department of Haematology, ASST Spedali Civili di Brescia, 25123 Brescia, Italy; alessandra.tucci@asst-spedalicivili.it; 5 Faculty of Biomedical Sciences, Università della Svizzera Italiana (USI), 6900 Lugano, Switzerland; giorgio.treglia@eoc.ch; 6 Department of Nuclear Medicine and Molecular Imaging, Lausanne University Hospital, University of Lausanne, 1011 Lausanne, Switzerland; 7 Clinic of Nuclear Medicine, Imaging Institute of Southern Switzerland, Ente Ospedaliero Cantonale, 6500 Bellinzona, Switzerland; 8 San Raffaele Scientific Institute, 20132 Milan, Italy; chiti.arturo@hsr.it (A.C.); fallanca.federico@hsr.it (F.F.)

**Keywords:** PET/CT, PET, nuclear medicine, ENKTCL, lymphoma, molecular imaging, positron emission tomography, hybrid imaging, lymphoproliferative disorders, extranodal lymphoma

## Abstract

**Background/Objectives:** Extranodal NK-/T-cell lymphoma (ENKTCL) is a rare and highly aggressive lymphoma with a bad prognosis. The aim of our analysis is to evaluate existing research on the potential usefulness of fluorine-18-fluorodeoxyglucose positron emission tomography or positron/computed tomography (2-[18F]FDG PET or PET/CT) in the management of patients with ENKTCL. **Methods:** A complete search of the literature was conducted across Scopus, PubMed/MEDLINE, and Embase databases, focusing on articles published up to March 2025. **Results:** A total of 21 studies that investigated the role of 2-[18F]FDG PET or PET/CT in ENKTCL were included in our analysis. The main findings from the literature analysis were (1) 2-[18F]FDG PET/CT seems to be helpful in staging settings, showing a better diagnostic performance than conventional imaging and a positive impact on clinical stage; (2) 2-[18F]FDG PET/CT had excellent negative predictive value for detecting bone marrow involvement, especially in early-stage disease; and (3) qualitative and semiquantitative PET parameters might predict prognosis. **Conclusions:** Despite several limitations affecting this analysis, especially related to the heterogeneity of the studies included, 2-[18F]FDG PET/CT seems to be a useful tool for the evaluation of ENKTCL.

## 1. Introduction

Extranodal NK-/T-cell lymphoma (ENKTCL) is a rare and highly aggressive lymphoma that shows worthy racial and geographic differences worldwide. These lymphomas are mainly extranodal and are characterized by significant vascular damage and tissue destruction, with “nasal-type” as the most frequent localization [1]. In the pathogenesis of this cancer, Epstein–Barr virus (EBV) is thought to play a crucial role. Immunohistochemistry tests, including EBV-encoded small RNA in situ hybridization, are fundamental in the diagnostic process [2,3]. Progress has been made in understanding the disease; in the mid-1990s, it was demonstrated that ENKTCL production of P-glycoprotein was due to the expression of the MDR1/ABCB1 gene. Additionally, the apoptotic effect of asparaginase on NKTCL cell lines was shown in vitro. Despite the introduction of non-anthracycline-based chemotherapy regimens and the use of L-asparaginase, which have improved survival rates, the prognosis remains poor in cases of refractory or relapsed disease. For accurate staging of this disease, fluorine-18 fluorodeoxyglucose positron emission tomography (2-[18F]FDG PET) is a non-invasive essential tool due to the increased glucose metabolism and consequent increased radiopharmaceutical uptake of this aggressive lymphoma variant. For localized disease, a combination of chemotherapy and radiotherapy using non-anthracycline-based regimens is suggested, while for advanced stages, regimens containing L-asparaginase have shown improved survival outcomes, although relapsed and refractory cases have very poor survival [4]. Despite advancements in understanding the disease’s pathways, upfront treatment primarily involves chemotherapy and radiotherapy, and treatment-related mortality remains significant [5]. For this reason, an accurate and early diagnosis and staging might significantly impact patient outcomes. Moreover, a better approach to identifying high-risk patients seems to be essential. Due to the rarity of the disease, international collaborations and clinical trials are shareable to shape the future of ENKTCL diagnosis and treatment.

## 2. Materials and Methods

### 2.1. Protocol

The current systematic review was carried out following a preset protocol, and the “Preferred Reporting Items for a Systematic Review and Meta-Analysis” (PRISMA 2020 statement) served as a guideline for its development and reporting [6]. The complete PRISMA checklist is in the Supplementary Materials (Table S1). Pre-registering was not carried out. As a first step, a direct review query using the Population, Intervention, Comparator, and Outcomes (PICO) framework was conducted: “What is the role (“outcome”) of 2-[18F]FDG PET or PET/CT (“intervention”) in patients with ENKTCL (“population”) compared or not to other imaging methods (“comparator”)?”. Two investigators (D.A. and F.F.) independently performed the literature search, the study selection, the data extraction, and the quality evaluation. In case of disagreements, a third opinion (C.R.) was asked.

### 2.2. Search Strategy

A thorough search of the PubMed/MEDLINE, Scopus, and Embase databases was performed to identify relevant published articles regarding the use of 2-[18F]FDG PET or PET/CT in patients with ENKTCL.

Moreover, specific research on the ClinicalTrials.gov database for ongoing investigations (access date: 1 March 2025) was performed. We used a search algorithm based on a combination of the following terms: (1) “PET” OR “positron emission tomography” AND (2) “FDG” OR “fluorodeoxyglucose” AND (3) “ENKTCL” OR “natural killer/T cell”. Our research had no beginning date limit and was updated until 1 March 2025. Only articles in the English language were selected. To enlarge our research, references of the retrieved articles were also screened for searching additional papers. For the management of these articles, we used EndNote Basic 2025 (ThompsonReuters, Philadelpia, PA, USA).

### 2.3. Study Selection

Studies or subsets within studies that examined the utility of 2-[18F]FDG PET or PET/CT in patients with ENKTCL were considered eligible for inclusion. The exclusion criteria included (1) articles outside the relevant field; (2) review articles, meta-analyses, letters, conference proceedings, and editorials; and (3) case reports or small case series involving fewer than 10 patients with ENKTCL. Two researchers (F.F. and D.A.) independently screened the titles and abstracts based on these criteria and subsequently reviewed the full texts to assess their relevance. All studies included in the systematic review were incorporated into the meta-analysis if they provided sufficient data to evaluate the diagnostic performance of the test and if there was no risk of overlap with other studies from the same research group.

### 2.4. Data Extraction and Collection

To minimize potential biases, two researchers independently reviewed each study and extracted data from the full manuscripts, including figures and tables. For every included study, we gathered general information such as the first author, year of publication, country, study design, funding sources, number of participants, age, and gender. We also collected technical details like the type of scanner used, the radiopharmaceutical dose administered, uptake time, and image analysis methods. Additionally, data on the sensitivity, specificity, positive predictive value (PPV), negative predictive value (NPV), and overall accuracy of 2-[18F]FDG PET or PET/CT were extracted. The key findings from the included articles are summarized in the tables and detailed in Section 3.

### 2.5. Quality Assessment (Risk of Bias Assessment)

A quality assessment of the included articles was conducted to evaluate the risk of bias in each study related to the review question. Four areas—patient selection; the index test; the reference standard; and flow and timing—were examined for potential bias. Additionally, three aspects—patient selection; the index test; and the reference standard—were assessed for applicability concerns using the QUADAS-2 tool (Bristol, UK) [7].

### 2.6. Statistical Analysis

Diagnostic accuracy metrics were derived from each included study whenever possible, based on a per-patient analysis and bone marrow evaluation, using data such as true positives, false positives, true negatives, and false negatives. The main outcomes for the quantitative analysis were pooled sensitivity and specificity, calculated with a bivariate random-effects model that accounts for potential correlation between these two measures [8]. Additionally, the authors determined pooled positive and negative likelihood ratios (LR+ and LR−) and the diagnostic odds ratio (DOR). All pooled estimates were reported with 95% confidence intervals (95% CI). To provide a comprehensive overview of the diagnostic performance, a summary receiver operating characteristic (SROC) curve was also generated, illustrating the relationship between sensitivity and specificity [8]. If significant statistical heterogeneity was detected, subgroup analyses were planned, considering factors such as features of the index test, patient characteristics, technical aspects, and clinical scenarios. The presence and extent of heterogeneity were assessed using the I-squared (I^2^) index, with values over 50% indicating substantial heterogeneity [8]. For the statistical analysis, we used the open-source software OpenMeta Analyst^®^ (Brown University, Providence, RI, USA, version 10.12).

Concerning forest plots, they visually summarize the results of individual studies and the overall effect in a meta-analysis. They allow for quick assessment of the consistency in the direction and magnitude of the effect across studies. Each line represents a study, with a square indicating the effect size and a horizontal line showing the confidence interval. The size of the square reflects the study’s weight in the meta-analysis. A vertical line typically marks the line with no effect (e.g., odds ratio or risk ratio of 1). If most confidence intervals cross this line, it suggests inconsistency or non-significant results. In contrast, if the effect sizes are generally on the same side of the line and the confidence intervals do not overlap widely, this indicates consistency and potential significance. The presence of heterogeneity can also be assessed visually—wide variation in effect sizes and overlapping confidence intervals suggest heterogeneity; which is formally tested using statistical measures such as I^2^.

## 3. Results

### 3.1. Literature Search

**Figure 1:**
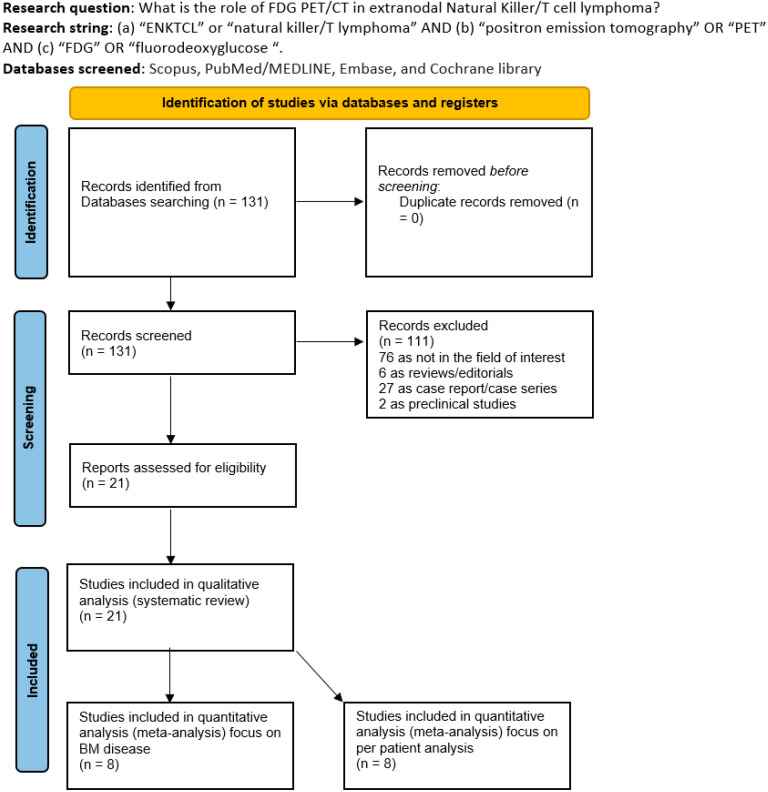
Literature Search flowchart.

During the literature search across the chosen databases, we identified a total of 131 records. After reviewing the titles and abstracts, 110 articles were excluded because they did not fall within the scope of our research—specifically; 76 were unrelated to the topic; 27 were small case series or case reports; 5 were reviews or editorials; and 2 were preclinical studies. Ultimately, 21 studies [9,10,11,12,13,14,15,16,17,18,19,20,21,22,23,24,25,26,27,28,29] were selected for full-text review and included in this systematic review (Figure 1).

No other articles were added after the revision of the references of the selected records.

### 3.2. Studies and Patients Data

The main features of the articles (all retrospective) included in the qualitative analysis were detailed in Table 1 and Table 2. The selected articles were published between 2007 and 2025, especially in China (*n* = 12), followed by Japan (*n* = 3), Korea (*n* = 3), and the USA (*n* = 3). Funding sources were reported only in five studies. The median/mean age ranged from 35.2 to 61 years, and males were more prevalent than females in all studies. The main purposes investigated in these studies were the diagnostic role of 2-[18F]FDG PET/CT in the staging (*n* = 10), bone marrow (BM) evaluation (*n* = 8), and the prognostic role of PET/CT (*n* = 8). In all studies except for 3, the scanner utilized was a hybrid PET/CT.

### 3.3. Risk of Bias and Applicability

**Figure 2:**
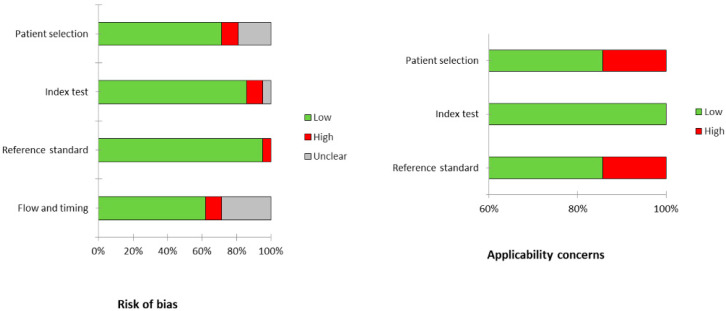
Representation of the QUADAS 2 score of the articles.

The overall assessment of the risk of bias and concerns about the applicability of the studies included in the systematic review, based on QUADAS-2, is summarized in Figure 2. The average radiotracer activity administered varied widely across studies. When expressed as relative values, the activity ranged from 3.5 to 4.5 MBq/kg, whereas in absolute terms, it varied from 185 to 740 MBq. The time interval between injection and scanning was approximately 60 min in all studies. PET images were evaluated qualitatively in all cases, and in 18 studies, a semiquantitative approach was also used. Among the semiquantitative parameters, the most commonly measured PET feature was the maximum standardized uptake value (SUVmax), followed by metabolic tumor volume (MTV), total lesion glycolysis (TLG), and texture features.

### 3.4. Staging

**Figure 3:**
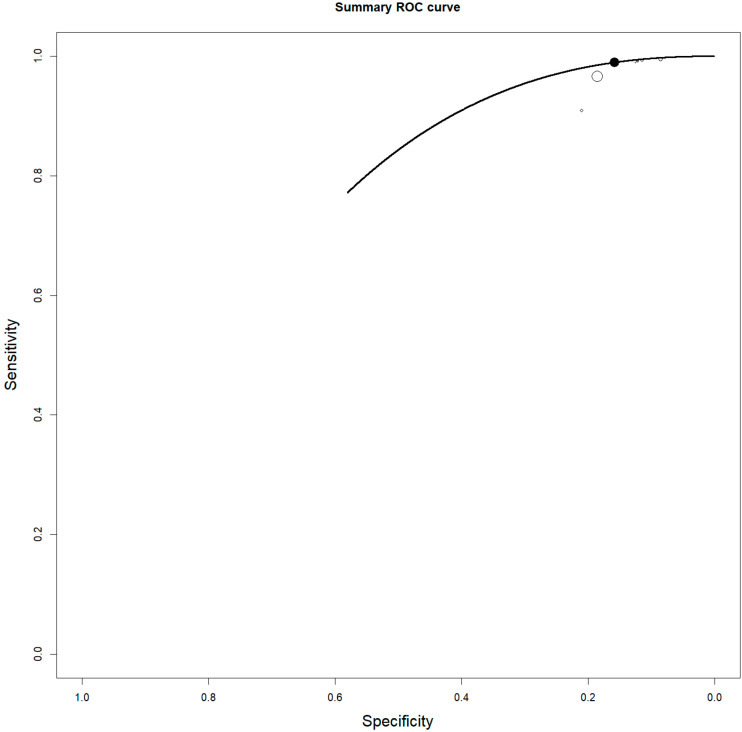
Summary ROC curve (SROC) on the staging performance of 2-[18F]FDG PET/CT in ENKTCL. Each circle represents a manuscript and the size of the circle represents the number of patients included.

**Figure 4:**
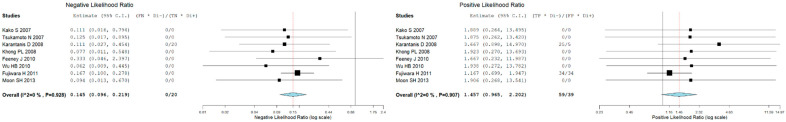
The figure displays the individual study results and the combined negative likelihood ratios (LR−) and positive likelihood ratios (LR+) of 2-[18F]FDG PET/CT in patients with ENKTCL, along with their 95% confidence intervals (95% CI). The size of each square reflects the weight assigned to that study. The horizontal lines represent the 95% CI for each individual study, while the width of the diamond indicates the 95% CI for the pooled LR− and LR+ [9,10,11,13,14,15,16,17].

**Figure 5:**
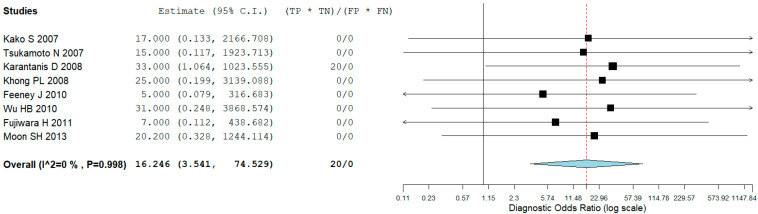
The plot illustrates the results of individual studies and the combined diagnostic odds ratio (DOR) of 2-[18F]FDG PET/CT in patients with ENKTCL, along with their 95% confidence intervals (95% CI). The size of each square indicates the weight assigned to each study. The horizontal lines represent the 95% CI for each individual study, while the width of the diamond reflects the 95% CI for the pooled DOR [9,10,11,13,14,15,16,17].

Eight studies, including 137 patients, were included in our bivariate patient-based meta-analysis [9,10,11,13,14,15,16,17]. The pooled sensitivity, specificity, PPV, NPV, and accuracy of 2-[18F]FDG PET or PET/CT were 97% (95% CI: 92–99%), 57% (95% CI: 18–90%), 95% (95% CI: 90–98%), 50% (24–76%), and 95% (95% CI: 90–98%), respectively (Table 3). The SROC curve is represented in Figure 3 and demonstrated a good diagnostic performance of 2-[18F]FDG PET/CT. The pooled LR−, LR+, and DOR were 0.145 (95% CI: 0.096–0.219), 1.457 (95% CI: 0.965–2.202), and 16.246 (95% CI: 3.541–74.529), respectively (Figure 4 and Figure 5). There was no statistically significant heterogeneity among the studies for any of the metrics assessed, as indicated by an I^2^ value of less than 50%.

### 3.5. Bone Marrow Evaluation

**Figure 6:**
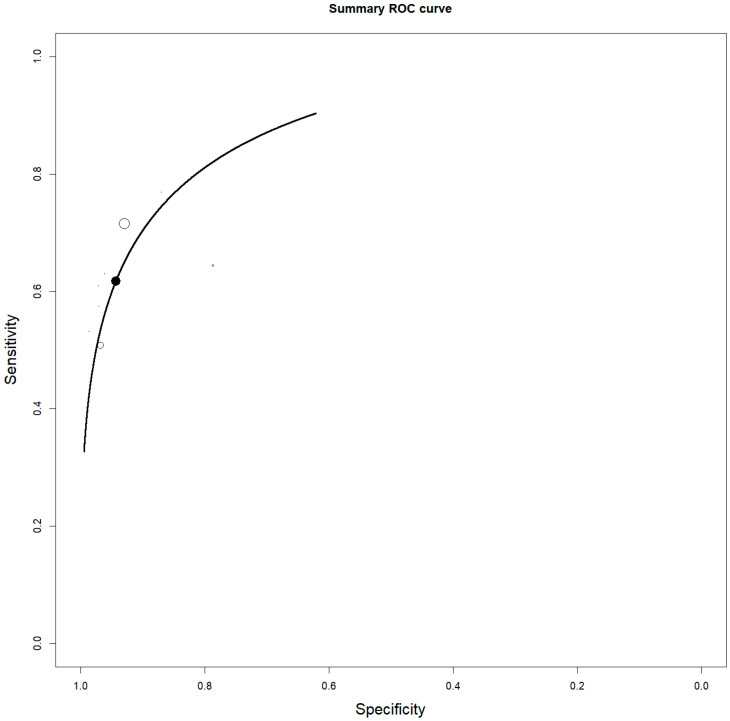
Summary ROC curve (SROC) on the performance of 2-[18F]FDG PET/CT in detecting BM disease in ENKTCL. Each circle represents a manuscript and the size of he circle represent the number of patients included.

**Figure 7:**
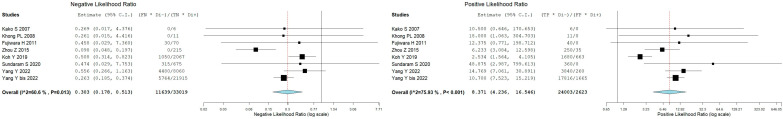
The plots display individual study results along with the pooled negative likelihood ratios (LR−) and positive likelihood ratios (LR+) of 2-[18F]FDG PET/CT in detecting bone marrow (BM) disease in ENKTCL patients, including 95% confidence intervals (95% CI). The size of the squares reflects the weight of each study in the overall analysis. The horizontal lines represent the 95% CI for each study, while the horizontal width of the rhombus indicates the 95% CI for the pooled LR− and LR+ [9,13,16,18,19,21,22,23].

**Figure 8:**
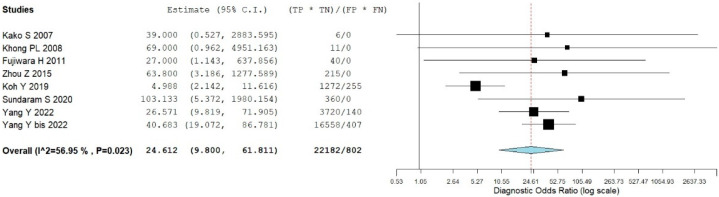
The plots display the results of individual studies and the combined diagnostic odds ratio (DOR) of 2-[18F]FDG PET/CT in detecting bone marrow (BM) disease in ENKTCL patients. They include 95% confidence intervals (95% CI). The size of each square indicates how much weight each study has in the overall analysis. The horizontal lines show the 95% CI for each study, while the width of the rhombus represents the 95% CI for the pooled DOR [9,13,16,18,19,21,22,23].

For the bivariate patient-based meta-analysis, 8 researches including 1175 patients were included [9,13,16,18,19,21,22,23]. The pooled sensitivity, specificity, PPV, NPV, and accuracy of 2-[18F]FDG PET or PET/CT were 64% (95% CI: 55–72%), 93% (95% CI: 91–95%), 56% (95% CI: 49–62%), 94% (95% CI: 94–96%), and 90% (95% CI: 88–91%), respectively (Table 4). The SROC curve is represented in Figure 6 and demonstrated a good diagnostic performance of 2-[18F]FDG PET/CT. The pooled LR−, LR+, and DOR were 0.303 (95% CI: 0.178–0.513), 8.371 (95% CI: 4.236–16.546), and 24.612 (95% CI: 9.800–61.811), respectively (Figure 7 and Figure 8). A significant heterogeneity among the studies was found for all the metrics evaluated (I^2^ > 50%).

### 3.6. Prognosis

Regarding the prognostic role of 2-[18F]FDG PET/CT in ENKTCL, eight studies are available in the literature, reporting a significant role of PET/CT features in predicting prognosis [12,20,24,25,26,27,28,29] (Table 5). First, Suh C. et al. [12], in a small population (*n* = 21), showed that SUVmax using a cutoff of 5.5 could predict poorer disease-specific survival. However, this cut-off was not confirmed by subsequent research that derived a higher value as the cut-off [28]. Also, the difference of SUVmax between baseline and interim PET/CT had a strong prognostic power, but also in this case, different thresholds were suggested [26,29]. Beyond semiquantitative variables, visual analysis also demonstrated a prognostic impact in this disease. Treatment response expressed by Deauville scores significantly predicted PFS and OS, especially Deauville score 5 [20,28]. Texture analysis was studied only in two papers [24,27] with promising results: deep learning models including PET features may help to stratify prognosis (PFS and OS).

## 4. Discussion

2-[18F]FDG PET/CT is a functional imaging test that has been widely used in the staging, prognosis, and treatment response valuation of Hodgkin lymphoma (HL) and various types of B-cell non-Hodgkin lymphomas (NHL) [30,31]. However, specific studies about the role of PET/CT in ENKTCL are relatively rare and have heterogeneous results. The value of 2-[18F]FDG PET/CT in evaluating ENKTCL is still under debate because it is not yet understood due to the low number of studies and patients evaluated in literature, in comparison with other more diffuse lymphomas, like HL, DLBCL, and FL. The first issue to answer is if this lymphoma is 2-[18F]FDG-avid or not. NKTCL is typically aggressive and may present with extranodal involvement, often affecting the nasal cavity, upper respiratory tract, or gastrointestinal system. Since this lymphoma is highly metabolically active, it tends to show up as 2-[18F]FDG-avid disease. In extranodal sites of the disease, lesions may present as small in size and with minimal density variation compared to the surrounding tissues; therefore, they are more easily detectable with PET than conventional morphologic imaging. In the literature, very few cases of false negative scans were reported, all described in one study [11]. In fact, the reported overall PPV of 2-[18F]FDG PET/CT is excellent (95%), while NPV is very low (50%). Also, the specificity is not so high (57%). Thus, the possibility of having false or negative findings is not low. Potential causes of false positive reports are inflammatory/infectious processes, while for negative findings, the size of the lesions or the incorrect patient preparation (high blood glucose level) are potential causes.

Only demonstrating a high detection rate, it is possible to use 2-[18F]FDG PET/CT for evaluating treatment response and prognosis. In case of a positive PET, the uptake is usually quite high, with an average value of SUVmax included between 5.2 and 16, and it seems that it is relatively higher in nasal cavities than extranasal [11]. Detection of BM disease in patients affected by lymphoma is crucial, but it may be challenging owing to sampling errors; it has been previously demonstrated that a third of patients will show unilateral positive BM that could have been missed [32]. Besides, the BM biopsy is considered the reference standard for assessment of BM involvement, but it has several limitations, like the invasive nature of the procedure, the unilateral sampling, the potential complications related to the procedure, and the very small part of BM studied sometimes not being representative of the real disease. For these reasons, we have controversial results in the literature about the role of 2-[18F]FDG PET/CT in BM evaluation in NHL and HL. Concerning DLBCL, a focal 2-[18F]FDG uptake in BM has to be considered a true positive report, avoiding BM biopsy, but a negative PET/CT cannot exclude with certainty BM involvement. For ENKTCL, the ability of 2-[18F]FDG PET/CT in studying BM involvement could be fundamental because BM disease significantly affects the staging and the treatment choice in this disease [33]. Globally, the accuracy of 2-[18F]FDG PET/CT in BM evaluation is good with very high NPV (94%), especially in early-stage patients, but with suboptimal sensitivity (64%) due to the presence of a non-negligible number of false negative reports. One of the limitations of the studies focused upon BM evaluation is the heterogeneity of study designs (I^2^ > 50%), including variations in disease sub-types that may have intrinsically different risks of BM involvement, stages, and interpretation criteria, causing a wide range of reported sensitivity (20–100%) and specificity (75–100%). Also, the definition of positive BM at PET/CT is under debate and needs deep investigation. For the evaluation of BM involvement in lymphoma on 2-[18F]FDG PET/CT, unifocal or multifocal increased uptake is consistently considered positive. However, the diffuse increased uptake pattern is controversial due to the difficulties in discriminating between abnormal and reactive diffuse tracer uptake. Thus it seems not so excessive to suggest avoiding BM biopsy in the case of negative 2-[18F]FDG PET/CT in early-stage patients, while PET may not replace BM biopsy in advanced-stage patients due to the potential risk of false negative findings and the complex histological features of bone marrow involvement in mature T- and natural killer-cell lymphomas [34].

Also, the cases of false positive findings on 2-[18F]FDG PET/CT reported are not so low, causing a moderate specificity. This is a strong risk to consider because the possibility of overstaging is not negligible and merits high consideration.

Considering ENKTCL as a 2-[18F]FDG-avid lymphoma, the ideal treatment response criteria based on PET/CT were Deauville criteria. Deauville scores were introduced for the evaluation of treatment response in 2-[18F]FDG-avid lymphoma, like HL and DLBCL [35], but their predictive and prognostic role was demonstrated also in other lymphoma variants, like Mantle Cell Lymphoma [36] and Burkitt Lymphoma [37]. In the two studies where the prognostic role of Deauville scores was evaluated [20,28], this criterion showed to be an independent prognostic factor. Especially patients with a Deauville score of 5 demonstrated having worse PFS and OS. The potential prognostic role of baseline 2-[18F]FDG PET/CT parameters remains an unresolved issue, with findings that are quite heterogeneous. Actually, almost all publications demonstrated a significant role of PET features in predicting the outcome, but the heterogeneity of the studies included is wide due to the different populations analyzed (for example, for age and stage), variations in sample size, and the kind of treatment. Additionally, the relatively small sample sizes and the use of different semiquantitative metabolic parameters (such as SUVs, ratios, MTV, and TLG) further complicate this analysis. For these reasons, the thresholds derived to stratify ENKTCL patients are different, reducing the potential application of these parameters in the clinical scenario [38]. In this context, the use of artificial intelligence systems could be a strong help.

An interesting point now investigated in the literature is the cost-effectiveness and prioritization in clinical practice of PET/CT in this scenario. Considering the difficulties to stage and investigate ENKTCL, PET/CT could reduce costs related to unnecessary procedures and/or useless treatments. However, well-designed studies with adequate follow-up need to be designed to answer this question. The widespread use in rural or low-income settings must be strategically prioritized. With thoughtful integration—including referral models; focused clinical use; and investment in infrastructure—PET/CT can deliver meaningful benefits without overwhelming limited healthcare budgets. Ultimately, its use should be guided by evidence-based protocols and cost-effectiveness thresholds tailored to local needs and capabilities.

Generally, the main limitation of the literature available about 2-[18F]FDG PET/CT and ENKTCL is the retrospective nature of all the studies included, which increases the risk of selection bias and missing data.

## 5. Conclusions

Despite the heterogeneity of the included studies, this systematic review and meta-analysis suggest that 2-[18F]FDG PET/CT may play a significant role in the evaluation of ENKTCL patients, particularly in disease staging.

It shows a notably high NPV in identifying BM involvement, especially in those with early-stage disease. However, its overall sensitivity remains low, which means the possibility of missing bone marrow lesions—i.e.; false negatives—should not be overlooked.

However, while preliminary findings regarding its prognostic value are promising, they remain inconclusive. Larger, prospective, multicenter studies are warranted to better define its potential role in the diagnostic workflow.

## Figures and Tables

**Table 1:** The main features of the studies selected.

First Author	Year	Country	Funding Source	Study Design	No. of ENKTCL Patients	M:F	Average Age (Range)	Purpose/s
Kako S [9]	2007	Japan	No	R	8	5:03	54 (43–73)	Staging, BM evaluation
Tsukamoto N [10]	2007	Japan	No	R	7	na	na	Staging
Karantanis D [11]	2008	USA	No	R	10	5:05	44.2 (27–60)	Staging
Suh C [12]	2008	Korea	No	R	21	14:07	46 (17–78)	Prognosis
Khong PL [13]	2008	China	No	R	12 *	8:04	50.6 (17–79)	Staging, BM evaluation
Feeney J [14]	2010	USA	No	R	12/122	11:01	na	Staging
Wu HB [15]	2010	China	No	R	15	11:04	42 (23–79)	Staging
Fujiwara H [16]	2011	Japan	No	R	19	11:08	61 (13–90)	Staging, BM evaluation
Moon SH [17]	2013	Korea	No	R	52	34:18:00	49.4 (24–74)	Staging
Zhou Z [18]	2015	China	No	R	55	37:18:00	44 (18–72)	BM evaluation
Koh Y [19]	2019	Korea	National Research Foundation of Korea (NRF) grant for the Global Core Research Center (No. 2011–0030001) and NRF grant for Research Center for Cellular Heterogeneity and Adaptation (No. NRF-2016R1A5A1011974) funded by the Korean government (Ministry of Science, ICT, and Future Planning).	R	46/109	67:42:00	55.2 (15–89)	BM evaluation
Qian L [20]	2020	China	No	R	15/51	27:24:00	35.2 (17–72)	Prognosis
Sundaram S [21]	2020	USA	No	R	Jul-60	34:26:00	58 ** (21–82)	BM evaluation
Yang Y [22]	2022	China	No	R	356	1.78:1	45 ** (13–77)	BM evaluation
Yang Y bis [23]	2022	China	No	R	742	531:211	na	Staging, BM evaluation
Luo Y [24]	2024	China	Medical Science and Technology Research Project of Henan Province (SBGJ202101002)	R	252	158:94	na	Prognosis
Zhu YM [25]	2024	China	National Key Research and Development of China (2020AAA0109504), National Natural Science Foundation of China (81970185), and the Fundamental Research Funds for the Central Universities (3332022028).	R	133	99:34:00	44 **	Prognosis
Ren Q [26]	2025	China	Youth Program, Natural Science Foundation of Hubei Province, People’s Republic of China (grant number 2022CFB713)	R	119	79:40:00	44 ** (18–77)	Prognosis
Jiang C [27]	2025	China	National Natural Science Foundation of China (grant 81971653), the 1·3·5 project for disciplines of excellence, West China Hospital, Sichuan University (grant ZYAI24014), China Postdoctoral Science Foundation (grant 2024M762244), and the National Natural Science Foundation of China (grant 82171975).	R	562	376:186	45 (35–55)	Prognosis
Liu L [28]	2025	China	No	R	133	82:51:00	46 ** (15–82)	Prognosis
Yang L [29]	2025	China	No	R	20	13:07	45 ** (30–71)	Prognosis

Legend: M: Male; F: Female; BM: Bone marrow; ENKTCL: Extranodal NK/T-cell lymphoma; R: Retrospective; P: Prospective; NA: Not available. * of 30 patients, ** median.

**Table 2:** The technical characteristics of studies included.

First Author	Device	Mean 2-[18F]FDG Injected Dose, MBq	Mean Uptake Time (min)	Image Analysis	Semiquantitative Variables
Kako S [9]	PET	296	60	Visual and semiquantitative analysis	SUVmax
Tsukamoto N [10]	PET	275–370	40–60	Visual and semiquantitative analysis	SUVmax
Karantanis D [11]	PET/CT	550–740	60–90	Visual and semiquantitative analysis	SUVmax
Suh C [12]	PET	555	60	Visual and semiquantitative analysis	SUVmax
Khong PL [13]	PET/CT	222–370	60	Visual and semiquantitative analysis	SUVmax
Feeney J [14]	PET/CT	555	60–90	Visual and semiquantitative analysis	SUVmax
Wu HB [15]	PET/CT	277–511	60	Visual and semiquantitative analysis	SUVmax
Fujiwara H [16]	PET/CT	3–4.3/kg	60–90	Visual and semiquantitative analysis	SUVmax
Moon SH [17]	PET/CT	5.5/kg	60	Visual and semiquantitative analysis	SUVmax
Zhou Z [18]	PET/CT	4.4/kg	60	Visual and semiquantitative analysis	SUVmax
Koh Y [19]	PET/CT	5.18/kg	60	Visual and semiquantitative analysis	SUVmax; MLR
Qian L [20]	PET/CT	na	na	Visual and semiquantitative analysis	SUVmax, DSUVmax
Sundaram S [21]	PET/CT	na	na	Visual analysis	
Yang Y [22]	PET/CT	5.18/kg	60	Visual analysis	
Yang Y bis [23]	PET/CT	na	na	Visual analysis	
Luo Y [24]	PET/CT	5.5/kg	60	Visual and semiquantitative analysis	SUVmax, MTV, TLG, texture analysis features
Zhu YM [25]	PET/CT	4–5/kg	60–70	Visual and semiquantitative analysis	SUVmax, SUVmean, MTV, and TLG
Ren Q [26]	PET/CT	5.55/kg	45–60	Visual and semiquantitative analysis	SUVmax
Jiang C [27]	PET/CT	185–370	60	Visual and semiquantitative analysis	SUVmax, MTV, TLG, texture analysis features
Liu L [28]	PET/CT	3.7/kg	60	Visual and semiquantitative analysis	SUVmax
Yang L [29]	PET/CT	3.7–5.55/kg	60	Visual and semiquantitative analysis	SUVlbm

Legend: na: Not available; SUV: Standardized uptake value; MLR: Marrow-to-liver ratio; LBM: Lean body mass; MTV: Metabolic tumor volume; TLG: Total lesion glycolysis.

**Table 3:** Diagnostic performance of 2-[18F]FDG PET or PET/CT in a per-patient analysis.

First Author	TP	FP	FN	TN	Median SUVmax
Kako S [9]	8	0	0	0	5.2 (2.1–13)
Tsukamoto N [10]	7	0	0	0	7.5 (4–14.5)
Karantanis D [11]	5	1	0	4	16 (5–25)
Khong PL [13]	12	0	0	0	8 (6.9–10)
Feeney J [14]	12	0	2	0	10.8 (4.9–23.3)
Wu HB [15]	15	0	0	0	10.01
Fujiwara H [16]	17	2	0	0	11.67 (3.73–41)
Moon SH [17]	50	0	2	0	na
Global	126	3	4	4	

TP: True positive; TN: True negative; FP: False positive; FN: False negative; na: Not available.

**Table 4:** Diagnostic performance of FDG PET or PET/CT for detecting bone marrow involvement.

First Author	TP	FP	FN	TN	Criteria for Positive PET	Standard Reference
Kako S [9]	1	0	0	6	focal uptake	BMB
Khong PL [13]	1	0	0	11	focal or diffuse uptake higher than liver/spleen	BMB
Fujiwara H [16]	4	0	3	10	focal uptake	BMB
Zhou Z [18]	5	7	0	43	focal or diffuse uptake higher than liver	BMB
Koh Y [19]	24	17	15	53	focal or diffuse uptake higher than liver	BMB
Sundaram S [21]	8	0	7	45	focal uptake higher than liver	BMB
Yang Y [22]	12	10	14	310	focal or diffuse uptake higher than liver	BMB
Yang Y bis [23]	34	37	11	487	focal uptake higher than liver	BMB
Global	89	71	50	965		

TP: True positive; TN: True negative; FP: False positive; FN: False negative; NA: Not available; BMB: Bone marrow biopsy.

**Table 5:** Summary of Studies Evaluating the Prognostic Role of 2-[18F]FDG PET/CT.

First Author	N° Patients	Parameters Considered	Endpoints	Main Findings
Suh C [12]	21	SUVmax	DSS	SUVmax > 5.5 was significantly associated with worse prognosis
Qian L [20]	51	DS at interim and ΔSUVmax	PFS and OS	DS significantly predict PFS and OS, ΔSUVmax significantly predict PFS
Luo Y [24]	126	texture analysis features	PFS and OS	deep learning model including PET features may help to stratify prognosis
Zhu YM [25]	133	SUVmax, SUVmean, MTV, and TLG with different thresholds	PFS and OS	all PET parameters were significantly associated with prognosis
Ren Q [26]	119	ΔSUVmax	treatment response after 2 cycles of chemotherapy, PFS, and OS	DSSTL ≥ 50% is associated with better prognosis
Jiang C [27]	562	texture analysis features	PFS and OS	deep learning model including PET features may help to stratify prognosis
Liu L [28]	133	DS, SUVmax, metabolic response on interim PET	PFS and OS	SUVmax (>9.2), DS 5, or with stable disease or relapsed/progressive disease associated with worse PFS and OS
Yang L [29]	20	ΔSUVmax baseline-interim PET	Treatment response at end of treatment	ΔSUVmax 66.75% may predict treatment response

DSS: Disease-specific survival; PFS: Progression-free survival; OS: Overall survival; DS: Deauville score; SUV: Standardized uptake value; MTV: Metabolic tumour volume; TLG: Total lesion glycolysis.

## Data Availability

The data presented in this study are available upon request from the corresponding author.

## Supplementary Materials

The following supporting information can be downloaded at: https://www.mdpi.com/article/10.3390/jcm14134582/s1, Table S1: PRISMA 2020 Checklist. Reference [6] is cited in the supplementary materials.

## Abbreviations

The following abbreviations are used in this manuscript:

ENKTCL	Extranodal NK/T-cell lymphoma
PET/CT	Positron emission tomography/computed tomography
18F-FDG	Fluorine-18-fluorodeoxyglucose
EBV	Epstein-Barr virus
PPV	positive predictive value
NPV	negative predictive value
LR	likelihood ratios
DOR	diagnostic odds ratio
SROC	summary receiver operating characteristic
I2	inconsistency index
SUV	standardized uptake value
MTV	metabolic tumor volume
TLG	total lesion glycolysis
BM	bone marrow
PFS	Progression-free survival
OS	overall survival
HL	Hodgkin lymphoma
NHL	non-Hodgkin lymphoma
DLBCL	diffuse large B-cell lymphoma
FL	follicular lymphoma
LD	Linear dichroism
